# Detection of iron deficiency anemia by medical images: a comparative study of machine learning algorithms

**DOI:** 10.1186/s13040-023-00319-z

**Published:** 2023-01-24

**Authors:** Peter Appiahene, Justice Williams Asare, Emmanuel Timmy Donkoh, Giovanni Dimauro, Rosalia Maglietta

**Affiliations:** 1 Department of Computer Science and Informatics, University of Energy and Natural Resources, Sunyani, Ghana; 2 Department of Basic and Applied Biology, University of Energy and Natural Resources, Sunyani, Ghana; 3 Coordinatore del Consiglio Di Interclasse Dei Corsi Di Studio in InformaticaDipartimento Di Informatica, Università Degli Studi Di Bari ‘Aldo Moro’, Bari, Italy; 4 Institute of Intelligent Industrial Systems and Technologies for Advanced Manufacturing, National Research Council, Bari, Italy

**Keywords:** Anemia, Image Augmentation, Machine learning algorithms, Red blood cell, Palpable palm, Region of Interest, Non-invasive

## Abstract

**Background:**

Anemia is one of the global public health problems that affect children and pregnant women. Anemia occurs when the level of red blood cells within the body decreases or when the structure of the red blood cells is destroyed or when the Hb level in the red blood cell is below the normal threshold, which results from one or more increased red cell destructions, blood loss, defective cell production or a depleted sum of Red Blood Cells.

**Methods:**

The method used in this study is divided into three phases: the datasets were gathered, which is the palm, pre-processed the image, which comprised; Extracted images, and augmented images, segmented the Region of Interest of the images and acquired their various components of the CIE L*a*b* colour space (also referred to as the CIELAB), and finally developed the proposed models for the detection of anemia using the various algorithms, which include CNN, k-NN, Nave Bayes, SVM, and Decision Tree. The experiment utilized 527 initial datasets, rotation, flipping and translation were utilized and augmented the dataset to 2635. We randomly divided the augmented dataset into 70%, 10%, and 20% and trained, validated and tested the models respectively.

**Results:**

The results of the study justify that the models performed appropriately when the palm is used to detect anemia, with the Naïve Bayes achieving a 99.96% accuracy while the SVM achieved the lowest accuracy of 96.34%, as the CNN also performed better with an accuracy of 99.92% in detecting anemia.

**Conclusions:**

The invasive method of detecting anemia is expensive and time-consuming; however, anemia can be detected through the use of non-invasive methods such as machine learning algorithms which is efficient, cost-effective and takes less time. In this work, we compared machine learning models such as CNN, k-NN, Decision Tree, Naïve Bayes, and SVM to detect anemia using images of the palm. Finally, the study supports other similar studies on the potency of the Machine Learning Algorithm as a non-invasive method in detecting iron deficiency anemia.

**Supplementary Information:**

The online version contains supplementary material available at 10.1186/s13040-023-00319-z.

## Background

Anemia is one of the global public health problems that affect children and pregnant women [1]. A study by WHO stated that 42% of children below the age of six and 40% of females who are pregnant worldwide are anemic [2, 3] which affects the world’s total population of 33%, as a result of iron deficiency [4].

Anemia occurs once the level of red blood cells within the body decreases or when the structure of the red blood cells is destroyed or weakened [5]. Anemia can also occur when the Hb level in the red blood cell is below the normal threshold, which results from one or more increased red cell destructions, blood loss, defective cell production or a depleted sum of Red Blood Cells [1]. Early detection of anemia helps to prevent irreversible organ damage [6].

Fatigue, weakness, dizziness and drowsiness, are some of the symptoms caused by anemia by which children and pregnant females are vulnerable, which vary within a country [7], with compounded risk of mortality for both mother and child. Iron deficiency anemia has additionally been shown to affect psychological features and physical development in children and reduce productivity in adults [8]. Long-term illness can also contribute to a patient’s risk of diagnosing anemia. Conditions that are associated with the complex occurrence of anemia include diabetes, kidney syndrome, cancer, HIV/AIDS, inflammatory bowel disease, and cardiovascular disease [9]. Malaria, bilharzia and hemoglobinopathies are other main contributors [2, 9]. Anemia can be categorized into many forms which comprise sickle cell, thalassemia, aplastic, iron deficiency, and vitamin or iron deficiency. Every type of anemia has its cause and can be temporary or long-term which ranges from minor to severe with several causes [4].

The non-invasive approach such as the use of machine learning algorithms is one of the procedures and methods used in detecting clinical diseases of which anemia detection cannot be left out in recent times [10]. Regards the invasive method of detecting anemia, which is costive, time-consuming, and painful to patients due to the extraction of blood and sometimes exposes clinicians to prick the cause of the blood extraction. The non-invasive approach is cost-effective, takes less time and is reliable through the use of the palm, conjunctiva, tongue and fingernails as compared to the invasive method, even though these human features can be used to detect anemia by assessing their paleness by medical officers, this is mostly left to the discretion of the physician or the health official [11]. In this study, we aim at using the pallor of the palm to detect anemia using machine learning algorithms through a comparative study of Decision Tree, SVM, Naïve Bayes, k-NN and CNN, since the palm is one of the essentials sites or features to detect anemia [12], particularly at the initial stage of the examination.

Many studies have been conducted with the use of non-invasive techniques such as machine learning algorithms in the detection of anemia mostly with the use of the conjunctiva of the eyes, though the palpable palm is quite less used in most studies as compared to the conjunctiva. With the use of images of the conjunctiva of the eyes and the palpable palm, the authors [13] classified anemia using the Naïve Bayes which resulted in an accuracy of 90%, while Chand et al*.* [14] affirmed that Palmer had an accuracy higher than that of the conjunctiva when assessed the efficiency of the palm, fingernails and the conjunctiva in anemia detection in children aged from two months to five years. Getaneh et al*.* [15] conducted a study on anemia detection using the pallor of the conjunctiva, tongue, nail bed and palm with the datasets from young children of 5 years and below. The outcomes of their work gave inference that the palm was able to achieve the utmost sensitivity of 58% in the detection of anemia, which was moderate through the combination of the conjunctiva and palm strengthened by a sensitivity of 73%.

For detecting anemia using a non-invasive method, in [6, 16] a Support Vector Machine (SVM) was used to computerized non-invasive which was easy and cost-effective and was developed by the authors for detecting anemia with the use of 19 datasets of images of the conjunctiva with hemoglobin levels known attaining an accurateness of 78.90% been 15 out of the 19 cases, while [16] considered how the algorithms for image processing and computer vision may be used to detect anemia from the photo of the consideration of the conjunctiva of the eyes with the helpfulness of the Least Square Support Vector Machine (LS-SVM). In the study conducted by [17] CNN was applied by the authors with the aid of segmented images of the conjunctiva to detect anemia and a sensitivity of 77.58% was achieved of which the results were compared with a test from the laboratory corresponding to the various image samples.

In addition, there was an anemia detection with the use of images of the conjunctiva whereas a new device was proposed for capturing the images centered on using the k-Nearest Neighbor (k-NN) algorithm which achieved a significant performance using non-anemic images and tested on 113 instances and proved to be substantial [8]. Likewise, the Decision Tree, Support Vector Machine and k-Nearest Neighbor were used in the detection of anemia by [18] with the use of pallor digital images in which the Decision Tree had the highest accuracy of 82.61% among the other models in performance. Similarly, Magdalena et al*.* [19], a Convolutional Neural Network utilized by the authors in their study to detect anemia achieved 94% accuracy when the Adam optimizer was used.

Regards to the related studies stated beyond it is a clear indication that non-invasive methods such as the use of machine learning algorithms are reliable in anemia detection due to their cost-effective, and timely results-oriented as the palm is one of the essential spots for anemia detection [12] using the non-invasive approach. However, since most of the studies used the conjunctiva, our study focuses on using the palm as the palm is one of the promising sites in anemia detection [15].

## Methodology

**Fig. 1:**
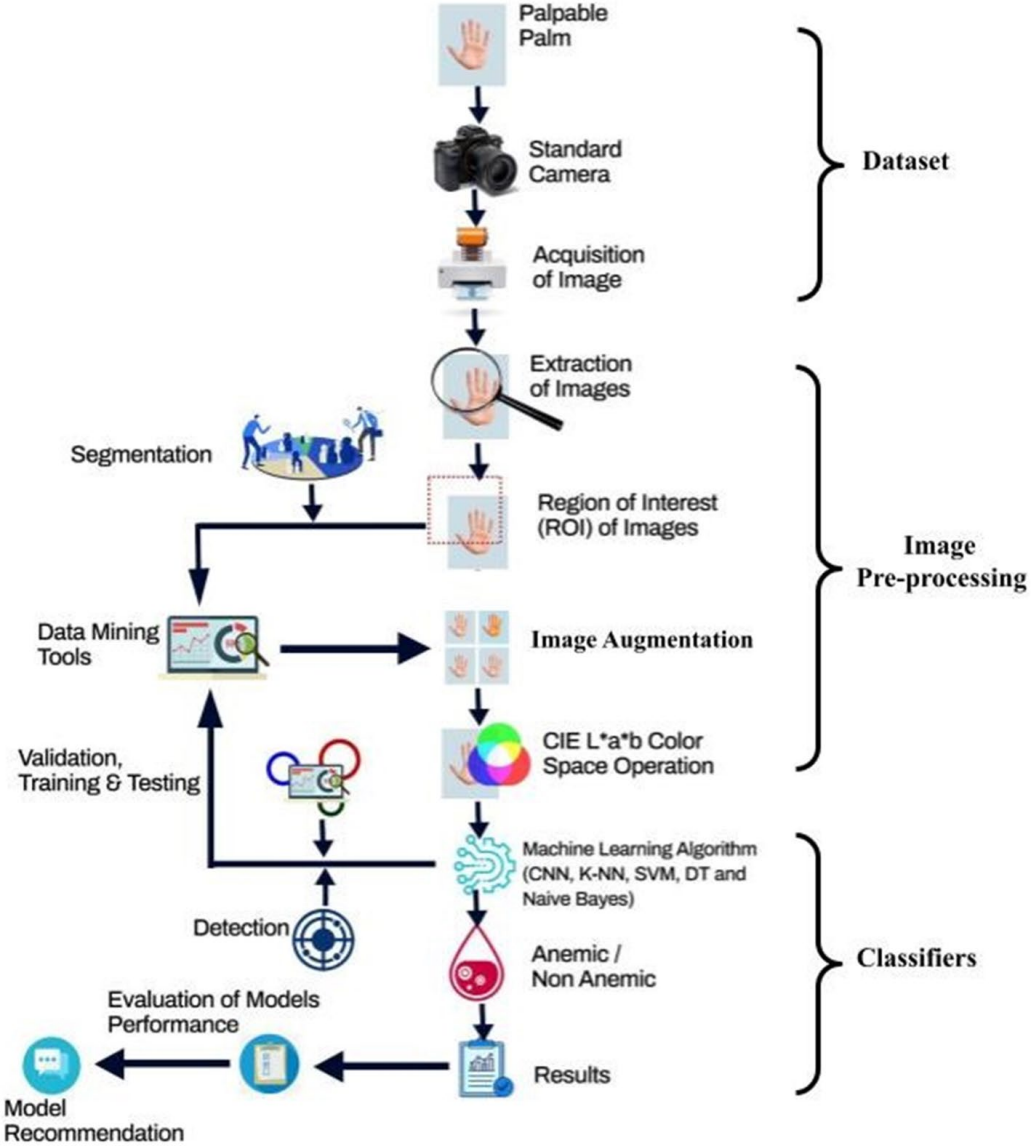
A Conceptual Framework of the Methodology

The technique or procedure employed in this study is explained and explored in this section, along with the numerous approaches used in the algorithms provided. This area is divided into three phases: datasets were collected, which is the palm, pre-processed the images, which comprised; Extracted images, augmented images, segmented the Region of Interest of the images and acquired their various components of the CIE L*a*b* colour space (also referred to as the CIELAB), and finally developed the proposed models for the detection of anemia using the various algorithms, which include CNN, k-NN, Nave Bayes, SVM, and Decision Tree (Fig. 1).

### Convolutional Neural Network (CNN)

Convolutional Neural Network (CNN) is a type of algorithm in machine learning that extracts and processes data in the form of pictures or images using classification [19]. CNN is composed of or contains two major components: feature extraction, which consists of distinct descriptions that aid in increasing the precision of the data to be processed and intends to extract vital information from the data, and the classification layer, which occurs after the extraction of data features via the use of fully connected neurons for transforming and the dimension of data [20, 21].

**Fig. 2:**
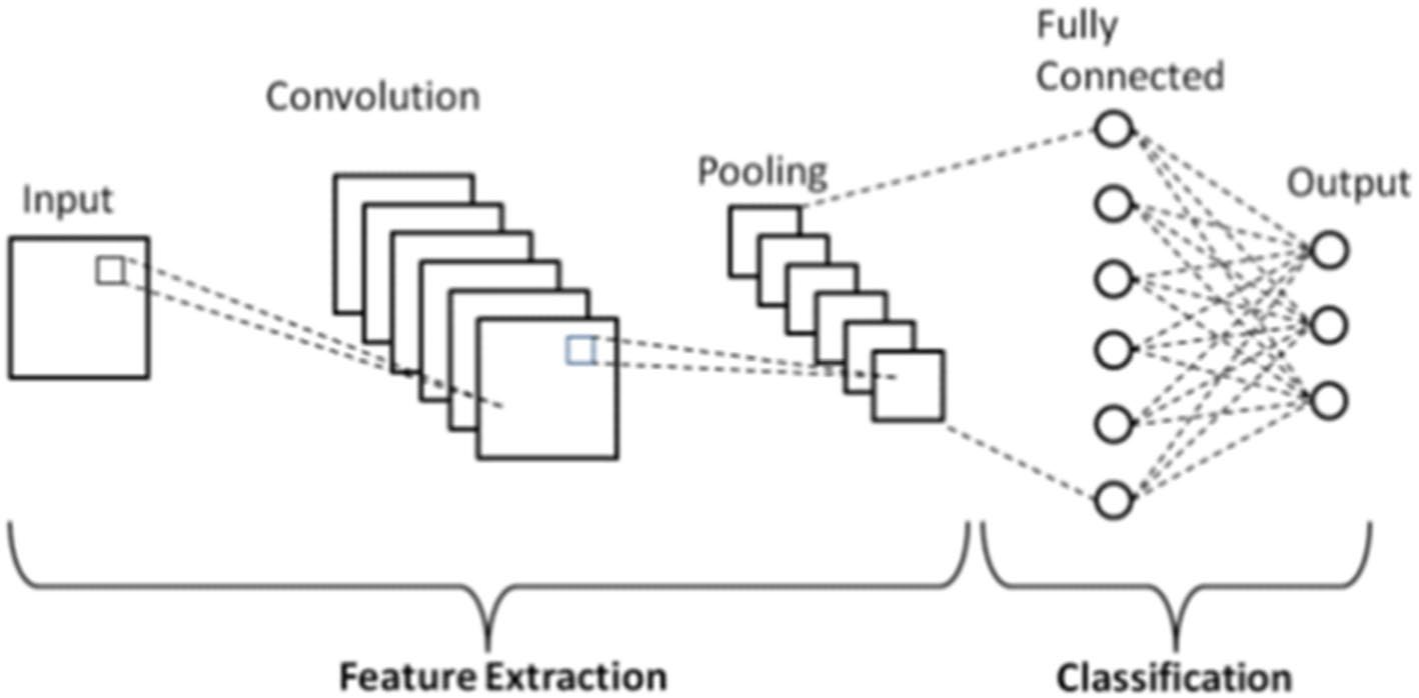
The architecture of the Convolutional Neural Network proposed model [19]

The CNN utilized the AlexNet and was trained with the Stochastic Gradient Descent (SGD) optimization and ReLu. The activation function used a regularization of $a=0.0001$, whiles the maximum iteration was set to 10. The prime function of the activation function is to convert the signal input of the nodes into a signal output. The CNN would become a linear regression with the absence of the activation function which would not be proficient to train the models which are complex (Fig. 2).

### Naïve Bayes

In Naïve Bayes, algorithm classifiers that are independent in assumptions are used, and the Bayes Theorem is implemented. The Naïve Bayes algorithm assumes that there is no relationship between the presence and absence of an attribute, and a small amount of training dataset is deemed fit for the calculation of the mean and variance of the variables associated [21] while splitting fields into discrete bin and target value fields. The Naïve Bayes usually generalize well since it has no hyperparameters to tune.

### Decision tree

**Fig. 3:**
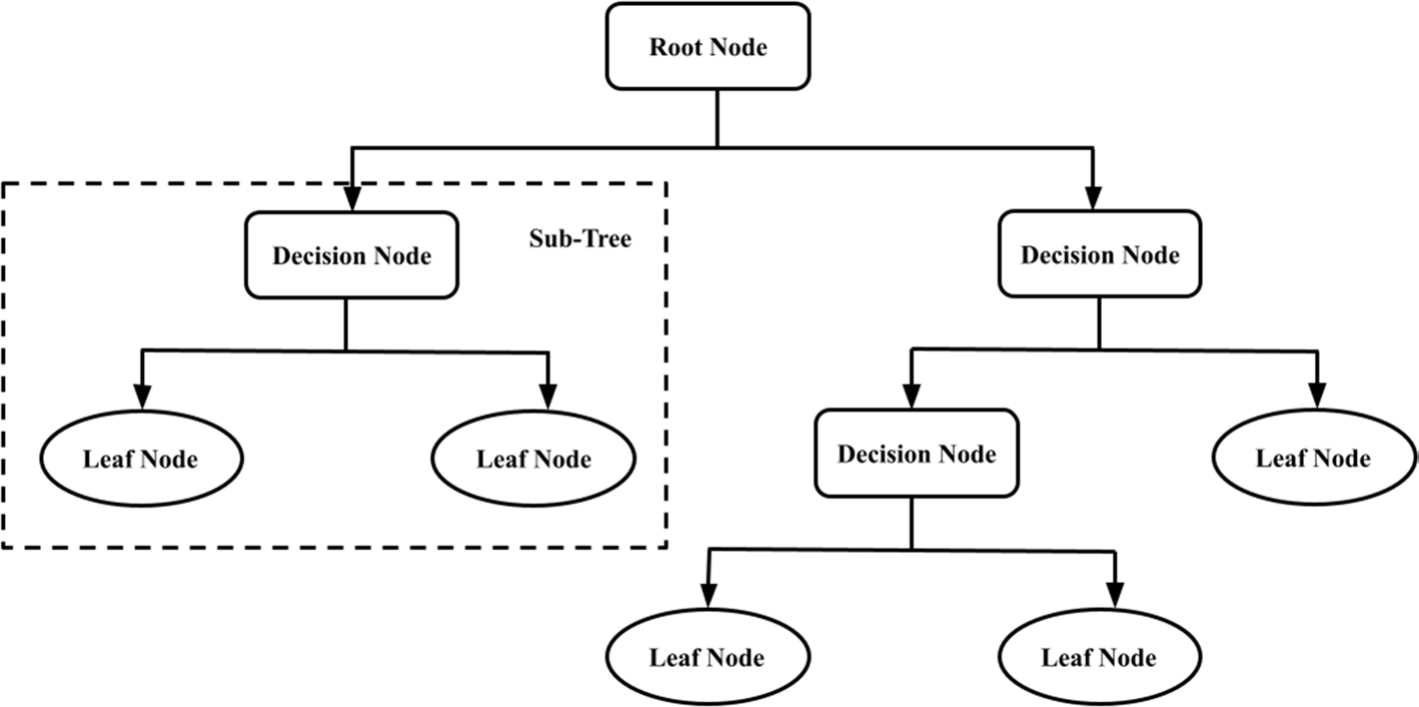
Decision Tree architectural diagram

The Decision Tree algorithm characterizes datasets with the use of a structured tree, which is employed in the computation of discrete target-valued functions. The categorization is accomplished by ordering the instances of the tree from some root to the leaf node [22]. Each branch of the tree signifies the value of each attribute, whereas the attribute denotes each node of the tree. The minimum number of instances in the leave was set to 10 and a minimum of 100 trees, while smaller subsets of less than 5 were not split and were used as the limit in the binary tree (Fig. 3).

### k-Nearest Neighbor (k-NN)

When comparing training, validation, and testing datasets, the k-NN method identifies comparable data [22]. The k-NN contains a tiny positive integer denoted by "k." In an experimental setting, a decision with a majority of neighbors is employed. An ideal case is when *k* = 2 [20] is allocated to the nearest neighbor in its class. The approach computes the distance between the feature vectors and their nearest neighbors and does not generate duplicates, instead producing synthetic data points that varied slightly from the actual data points. The k-NN had 100 neighbors as the metric was set to Euclidean with uniform weight [7, 23].

### Support Vector Machine (SVM)

**Fig. 4:**
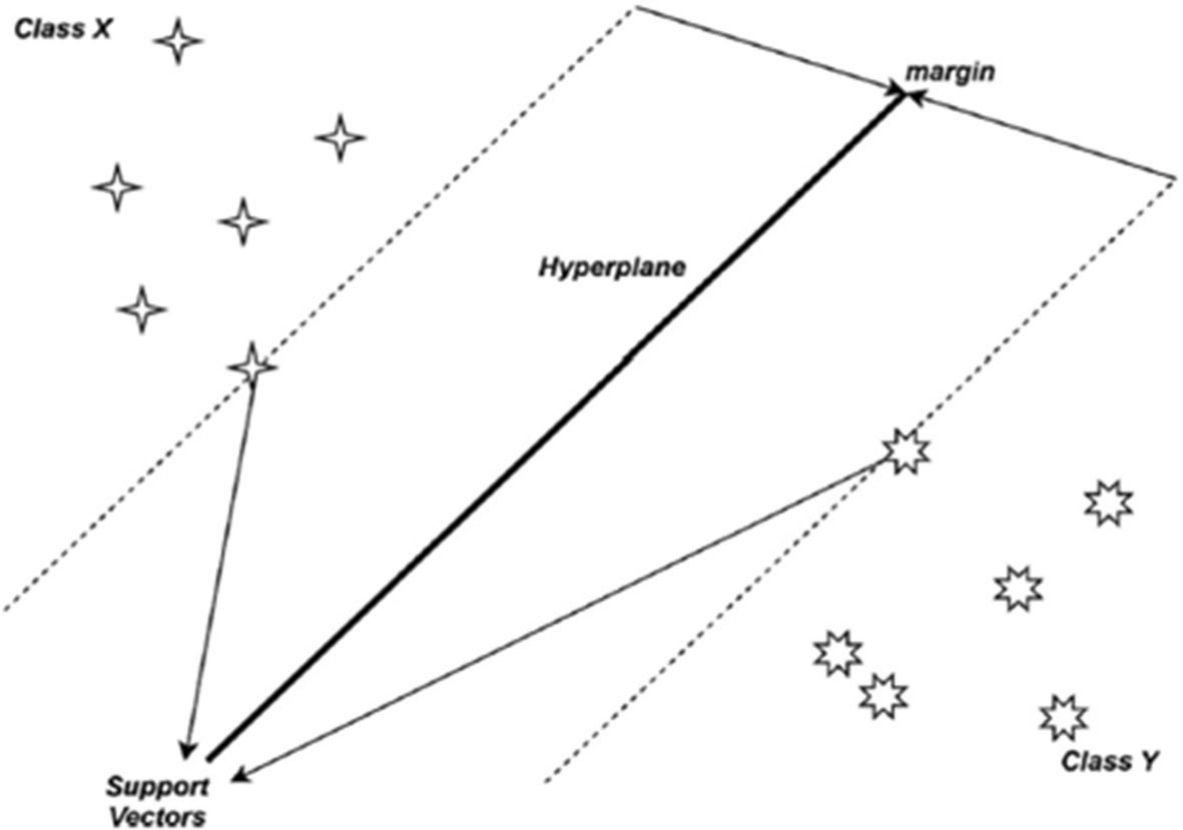
The proposed model Architectural Structure for the SVM [25]

Support vector machines (SVMs) are relatively new and widely used classification tools in which many components are integrated from previously used approaches. SVMs, like discriminant analysis, are based on the assumption that data is "separable" which implies it may be broken into groups by a functional separator [24]. Furthermore, SVMs are founded on the statistical learning theorem and aggressively expand this notation of separability centered on multiple concepts. The distinction of items due to the decision plane corresponds to separate types [22]. With the SVM, the Sigmoid was used for the operation with 100 iterations as the limit, cost $\left(c\right)=100$ and epsilon of regression ($\varepsilon ) was$ assigned to 1.10 while the numerical tolerance was set to 0.1000 (Fig. 4).

### Data collection system and dataset

**Fig. 5:**
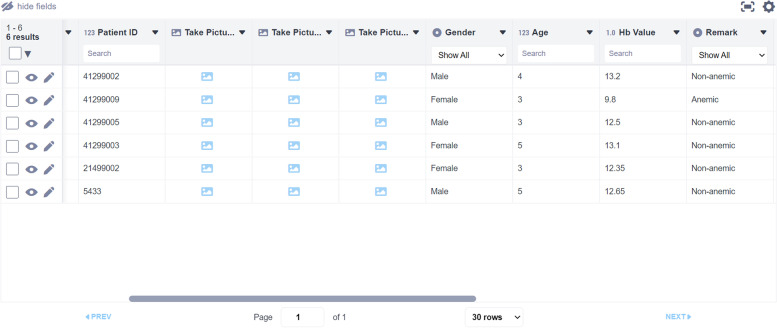
Interface of the database used during the data collection

**Fig. 6:**
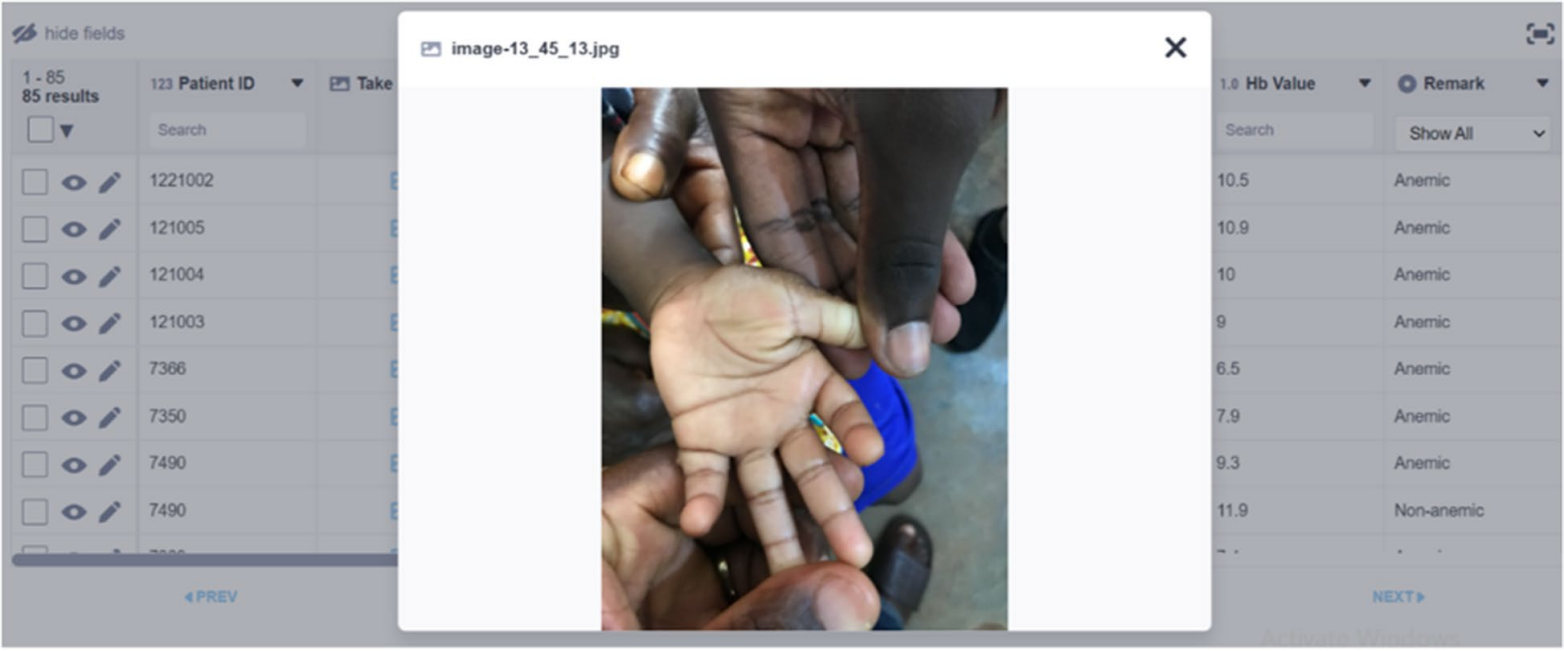
How the datasets were displayed in the database after they were selected for download

We established a data collecting system and trained medical laboratory staff on how to use the “ODK Collect” and “Kobo Collect” apps to collect the dataset (Fig. 5). A form was designed to collect patients' information such as Hb Value, age, gender, and a remark centered on the Hb Value obtained during the laboratory test, as well as a photo of the image of the palm to be uploaded to the database for easy access. All photographs were captured by laboratory technicians or medical laboratory officials using a typical high-quality camera with a minimum resolution of 12MP. Because the participants were children, the health officer(s) would grasp the hand and extend the palm through the fingers before photographing the palm as indicated in Fig. 6 below. Furthermore, to minimize inflated shine effects caused by the picture quality, which greatly impacts the detection or classification by the models, the cameras' spotlights were turned off when photographing the photos. This approach is an excellent way to eliminate the impact of ambient light on photos in datasets.

Following that, we used the triangle thresholding approach to extract the features of the palm images to generate the ROI, and then we utilized the image augmentation technique on the original datasets, which includes rotation, flipping, and translation, to expand the size of the original datasets, as small data size might lead to overfitting [3]. Following that, the extracted Region of Interest (ROI) is divided into metrics through the utilization of the CIE L*a*b* colour space intensity value of the pictures, where the CIE L*a*b* colour space value was denoted ${n}_{1},{n}_{{2}^{, }}{n}_{3},\cdots {n}_{n}$ signifying the different metric values. The datasets deployed in the study focus on children aged 5 and under, since the World Health Organization research indicates that 42 percent of young children aged 5 and under are anemic [26, 27].

The datasets used for this study were gathered from the following hospitals; Komfo Anokye Teaching Hospital at Kumasi, Bolgatanga Regional Hospital at Bolgatanga, Kintampo Municipal Hospital at Kintampo, Ahmadiyya Muslim Hospital at Techiman, Sunyani Municipal Hospital at Sunyani, Manhyia District Hospital at Kumasi, Ejusu Government Hospital at Ejusu, SDA Hospital at Sunyani, Nkawie-Toase Government Hospital at Nkawie-Toase and Holy Family Hospital at Berekum, all situated in Ghana. We organized a training seminar for the medical laboratory personnel on how to use the “ODK Collect” and the “Kobo Collect” apps to collect the datasets and submit them to the database (Table 1).

**Table 1:** Images of the ROI of patients' palms correlating to data after ROI extraction

**Patient ID**	**Palpable Palms (ROI)**	**Hb Value (g/ml)**	**Age**	**Sex**	**Remark**	
					**Anemic**	**Non-anemic**
PID-001		8.10	3	Female	Anemic	
PID-002		11.90	3	Male		Non-anemic
PID-003		10.40	2	Male	Anemic	
PID-004		12.30	4	Female		Non-anemic

### Data preprocessing

Depending on the outcome of image preprocessing and the development of the dataset, each image's ROI is converted in the CIELAB (also known as CIE L*a*b*) colour space model. The L*a*b colour space is intended to simulate human eyesight or perception. The standard deviation value of the ROI a* components, which is the mean value, is used to express it. A* components are red components (a* > 0) and green components (a* 0). The average value A* components are red components (a* > 0) and green components (a* 0). Previous research in this field shows that there is a strong relationship between a* components and Hb levels when calculated using the Pearson Correlation Index, and various experiments in this domain show that individuals with higher Hb values tend to have an average value of a* greater than 160, while patients with lower Hb values tend to have an average value of a* less than 142 [22].

As a result, the average values of the a* components appear to be more discriminating (that is, the mean intensity of red and green components better differentiates anemic and non-anemic individuals). The datasets are analyzed to get the detection of anemia. To begin, colour characterization of pictures is performed here utilizing the CIE L*a*b* colour space. This colour space converts all colours visible to the human eye into a three-dimensional integer space, allowing device-independent digital representation. Because the aforementioned components correlate to systemic changes in the alleged colour, the relative perceptual differences between two colours in L*, a*, and b* may be determined. Each colour may be approximated by considering it as a point in three-dimensional space (with three components: L* a*b*) and measuring the Euclidean distance between them. L* (Lightness) indicates the darkest black at zero and the brightest white at one hundred, whereas a* and b* are colour channels. Nonalignment grey is defined by its existence in the Cartesian coordinate system (a*, b*).

**Fig. 7:**
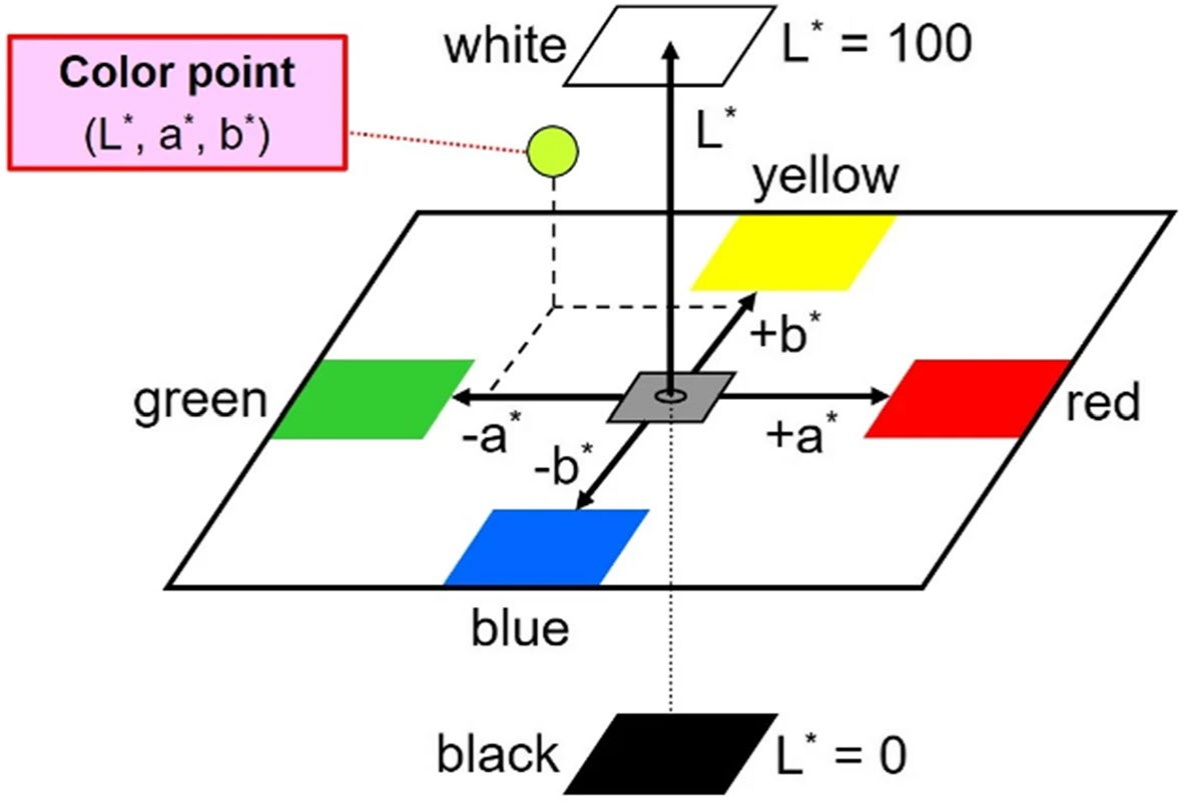
The Three-Dimensional CIE L*a*b* Colour Space Diagram (Beetsma, 2020)

**Fig. 8:**
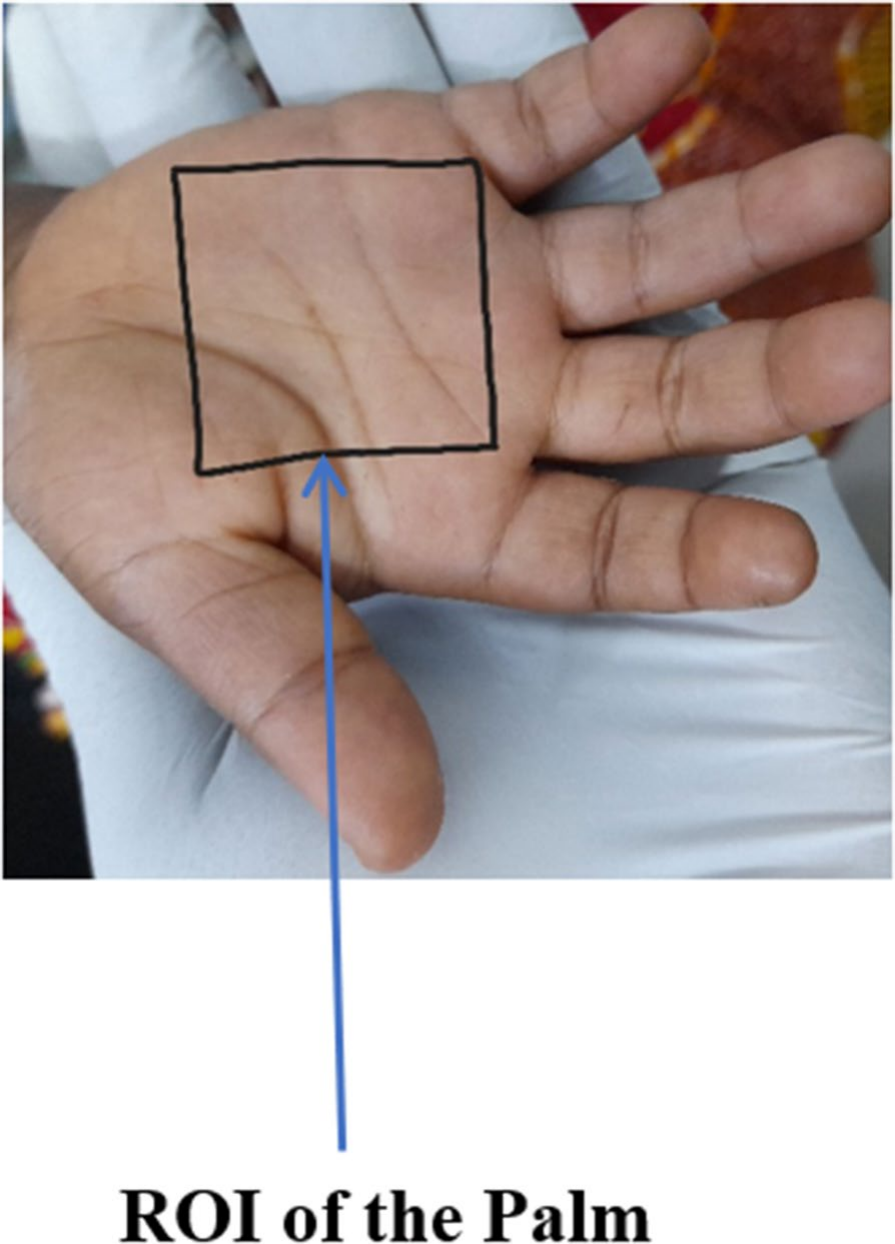
ROI of the palm

**Fig. 9:**
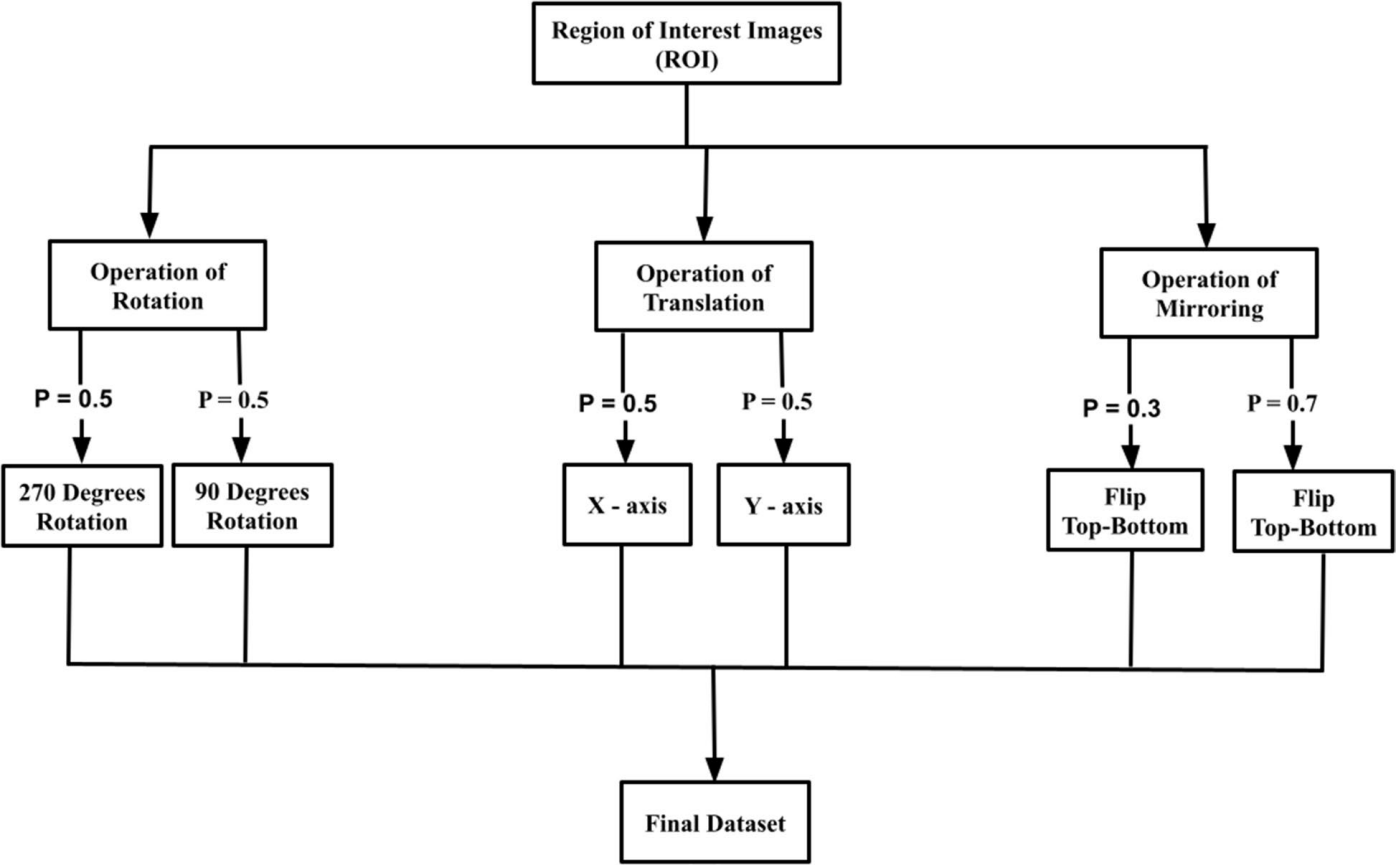
Diagram of Image Augmentation Technique used for the study as *p* = probability [28]

The colours of the opponent are portrayed on the b* axis, with yellow at b* > 0 and blue at b* 0. The colours of the foe are depicted on the a* axis, with red at a* > 0 and green at a* 0. The best classifying result was reached after numerous trials by integrating three-component features in total, namely a*, b*, and the G value derived from the RGB component photos, mapping the RGB to CIELAB colour space (RGB → CIELAB). The L and RGB component values were retained for filtering the incoming data. The L and RGB component values were retained for filtering the incoming data. The mean values of the characteristics a*, b*, and G were carefully calculated by considering just the image pixels with minL < L < maxL and min < R = G = B < max. This last filtering guarantees that image pixels that are abnormally dark or bright are deleted, therefore the technique shown here takes into account only pixels that enable an accurate pallor evaluation of the photos (Figs. 7, 8 and 9).

### Image augmentation of the palm

**Fig. 10:**
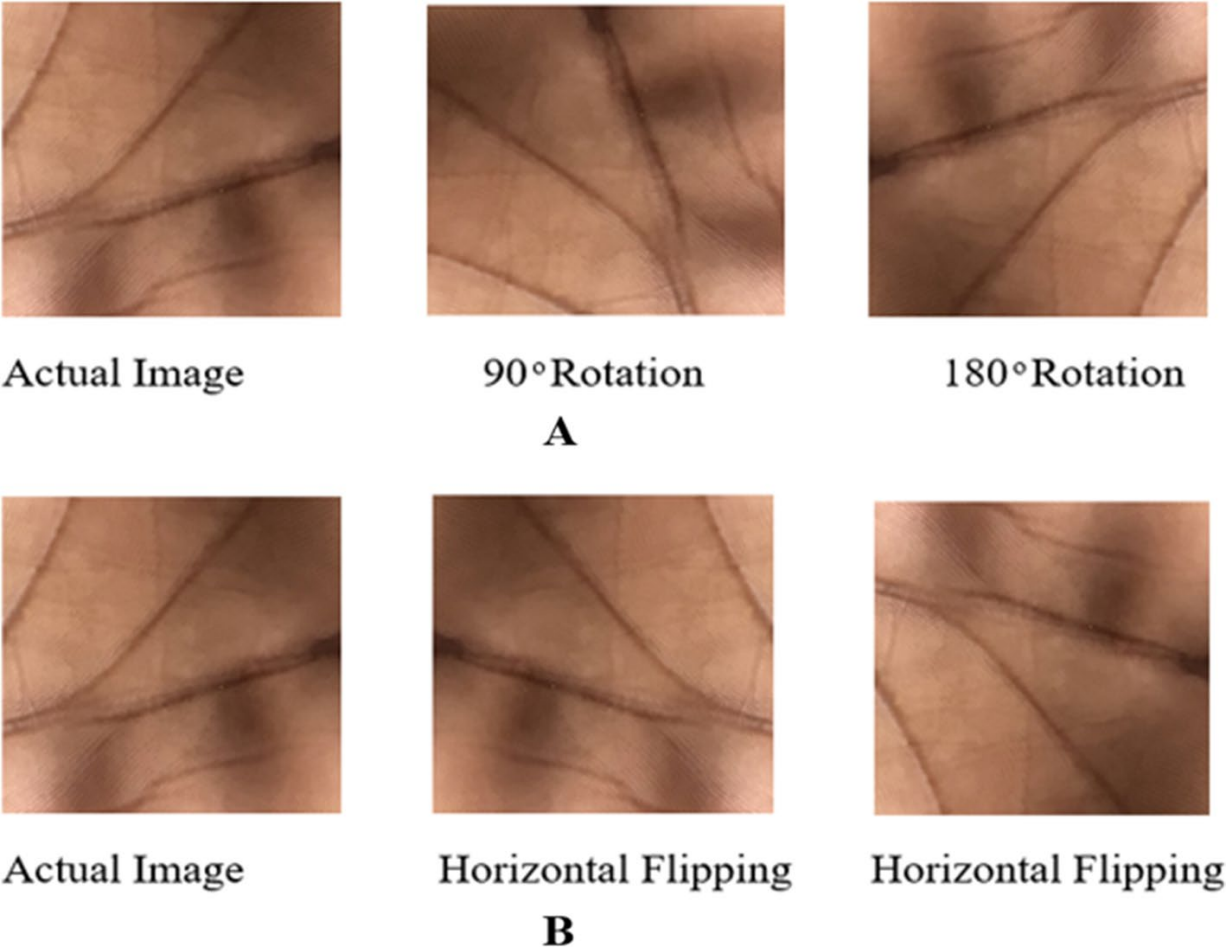
Diagram mage augmentation used for this study, **A** for Rotation and **B** for Flipping

Image augmentation techniques or methods were employed to expand the size of the datasets with the rationale to avoid overfitting by the models when used with small-size datasets for training [28]. Because datasets of anemia are complex to get, we employed rotation, flipping, and translation in this work to enhance the amount of the datasets. Cropping and Gaussian augmentation were not employed since they change the mean intensity of the picture components when used [28]. Figure 10 depicts some of the augmentation techniques used on the images and how they were applied, with (A) indicating rotation and (B) indicating flipping. In the process of augmenting the images to increase the size of the datasets, rotations of 90 degrees, and 270 degrees were applied to the original datasets, as well as flipped or (mirrored/mirroring) using the vertical and horizontal methods, and finally a translation to the X and Y axes also on the original datasets. All images were augmented before the models were trained, validated and tested.

### Training, validation and testing

The approach of random sampling technique after data augmentation to the entire dataset of 2635 as mentioned in Table 2 above was applied for training, validating, and testing the models using 70% for training, and 10% for validation by using a tenfold cross-validation, and 20% for testing (Table 3). Table 4 shows how the datasets were divided for training, validation, and testing.

**Table 2:** After preprocessing, a detailed description of the original and augmented dataset

	Anemic	Non-anemic	Total Dataset
Original Datasets	304	223	527
Augmented Datasets	1520	1115	2635

**Table 3:** Statistics on the original dataset and the Hemoglobin concentration level (g/dL)

	**Patient Class**			
	**Anemic**		**Non-Anemic**	**Total**
Patients	304 (58%)		223 (42%)	527 (100%)
Female	180 (60%)		118 (40%)	298 (57%)
Male	124 (54%)		105 (46%)	229 (43%)
	**Anemia Diagnosis for Age 6–59 months (6 months to 5 years)**			
Anemia Classification	Anemic	Non-Anemic		
Hb Levels	< 11 g/dL	> = 11 g/dL

**Table 4:** A detailed description of how the datasets were used (trained, validated and tested) in percentage

	**Palm**		**Total**
	Anemic	Non-anemic	
**Training Dataset (70%)**	1064	781	**1845**
**Validation Dataset (10%)**	152	111	**263**
**Testing Dataset (20%)**	304	223	**527**
**Total**	**1520**	**1115**	**2635**

### Evaluation and performance measures

Evaluation of models is essential because it quantifies a classifier's performance as a generic model. This means that the input–output relationships developed from the training data set must also operate on the validation dataset [29, 30]. The most unique technique to assess the prediction accuracy of a Machine Learning classifier is to extensively test the classifier on a collection of independent samples to integrate all conceivable causes of variability to be encountered [29]. To measure the performance of the models, the results acquired by the various models were evaluated using evaluation matrices such as precision, recall, F1-score, and AUC. Whiles a tenfold cross-validation was used in validating the datasets.

1 $$\mathrm{Specificity }=\frac{TN}{TN+FP}$$

2 $$\mathrm{F}1-\mathrm{Score }= \frac{2(P.R)}{P+R}$$

3 $$\mathrm{Accuracy }= \frac{TP+TN}{TP+TN+FP+FN}$$

4 $$\mathrm{AUC }= \frac{TPR-TNR}{2}$$

5 $$\mathrm{Recall }= \frac{TP}{TP+FN}$$

6 $$\mathrm{Precision }= \frac{TP}{TP+FP}$$

where the True Positive number of samples after detection, and the valid values are positive are denoted by TP, the True Negative number by TN, the False Negative number by FN, the Precision, the Recall, and the True Negative Rate by TNR.

## Results and discussions

The proposed models were designed to detect anemia by comparing several machine learning methods, notably CNN, k-NN, Naïve Bayes, SVM, and Decision Tree. The CNN was trained with the optimization and activation functions, as well as regularization of SGD and ReLu and a = 0.0001. The action function executes such actions for the node's signal input to be transformed to signal output, and the CNN becomes linear regression when the activation function is not used or applied. The sigmoid was utilized for SVM operations, with a limit of 100 iterations, a cost of (c) = 100, and an epsilon of regression ($\varepsilon$) of 1.10, with a numerical tolerance of 0.1000. We used several 100 for the neighbors with uniform weight, and the metric was set to Euclidean. The Decision Tree binary tree was induced with the minimum number of occurrences in the left set to 10. Also, we reduced subsets of less than five were not divided and minimal trees of 100 were set as the limit.

We used the orange data mining software for the experimental operation because the study by [27] yielded a significant result in the detection of liver disorder using Nave Bayes, k-NN, and Decision Tree, indicating that the orange data mining software is an efficient tool for the detection of medical diseases such as anemia and liver disorder. Also, Peker et al*.* (2018) employed Random Forest, ANN, k-NN, SVM, and Decision Tree Algorithms to diagnose and detect diabetes using the orange data mining software. The ANN obtained the best accuracy of 90.27%, while the SVM earned the lowest accuracy of 64.66% among the other methods. This demonstrates why orange data mining software is efficient and effective for detecting clinical or medical diseases [27, 31] including anemia, diabetes, and liver disease.

**Fig. 11:**
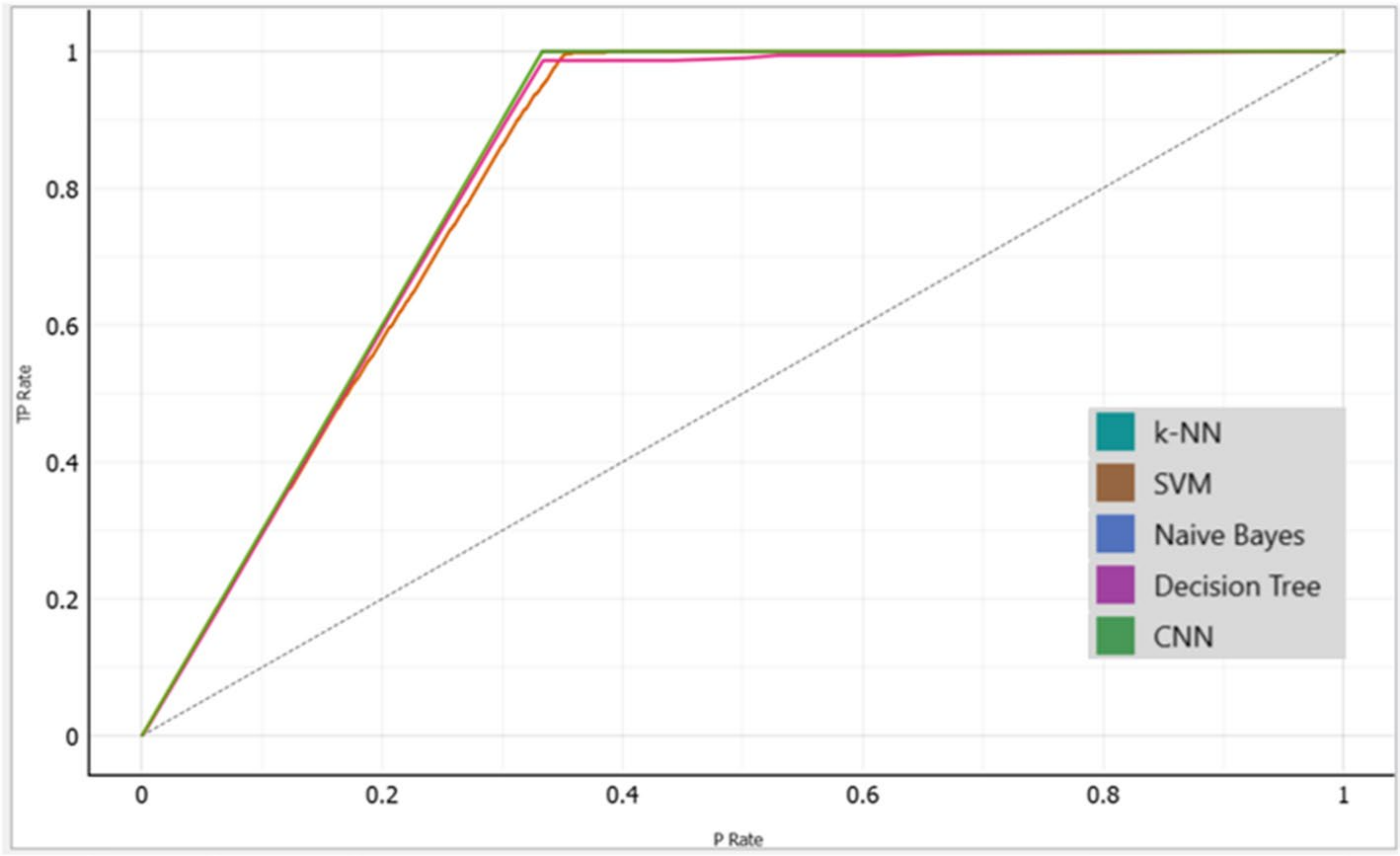
A graphical presentation of the Cumulative Gains of the Palm in anemia detection

**Fig. 12:**
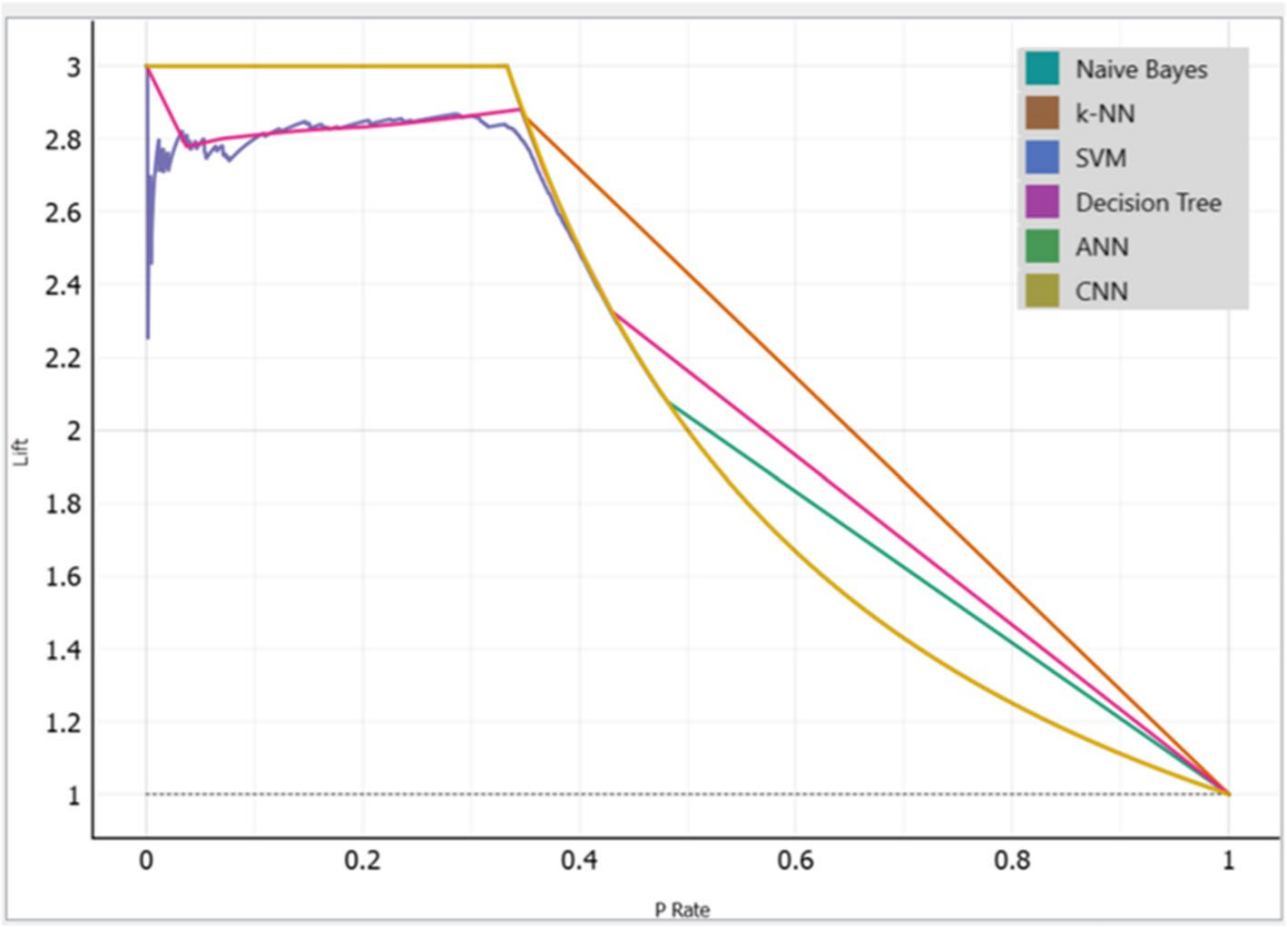
A graphical presentation of the Lift Curve of the Palm in anemia detection

**Fig. 13:**
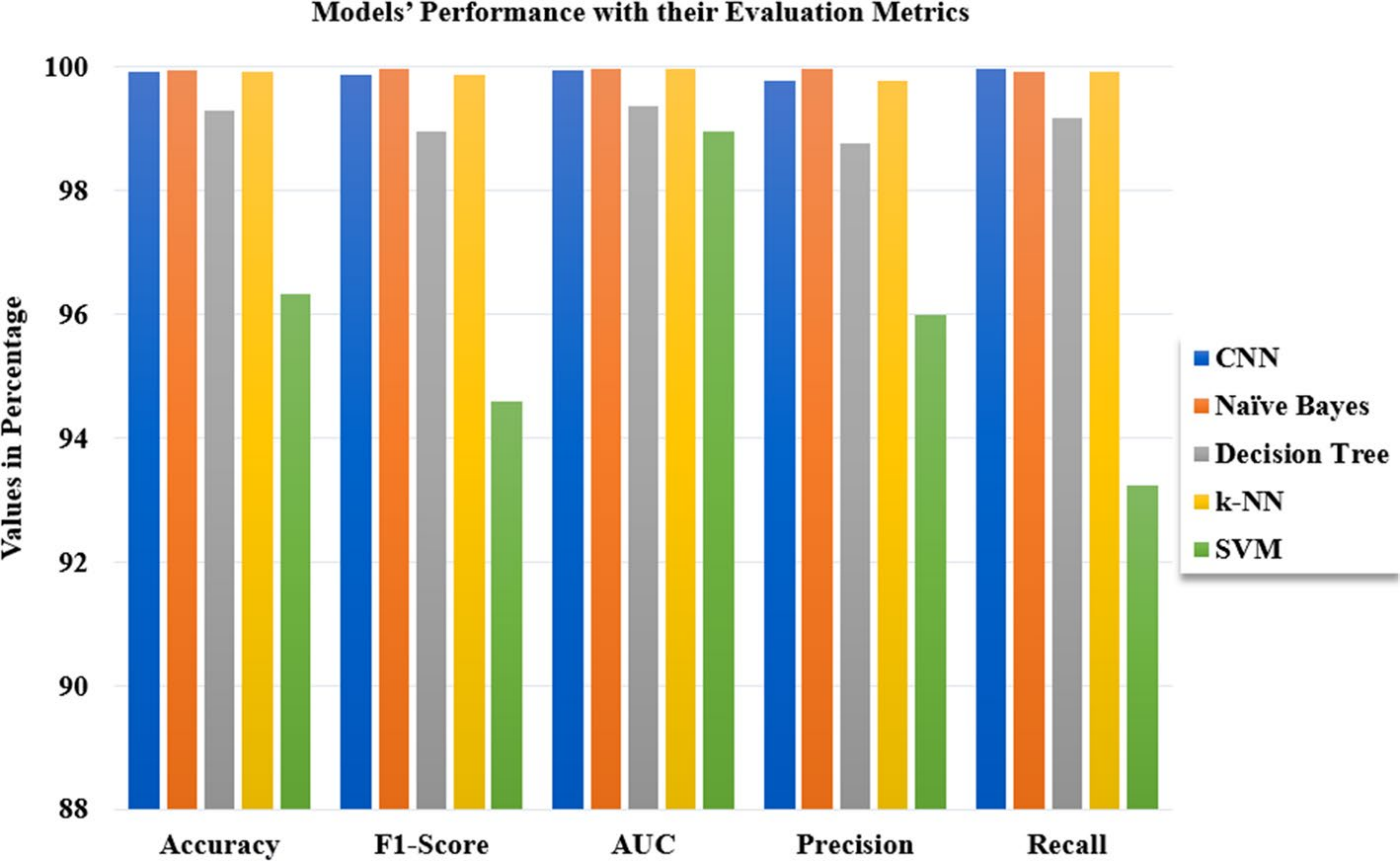
Graphical representation of models’ performance with their evaluation metrics

Following the training, validation, and testing of 2635 datasets, 1520 of which are anemic and 11,115 of which are not. Our proposed models performed well in the detection of anemia using images of the palm, with the Naïve Bayes achieving the highest accuracy of 99.96%, followed by the CNN and the k-NN with an accuracy of 99.92% achieved by each model, Decision Tree with an accuracy of 99.29%, SVM had the least accuracy of 96.34% among all models (Figs. 11, 12 and 13).

Table 5 shows the outcomes of all models together with their relevant evaluation measures, while Table 6 compares the results obtained in this study to the results of earlier studies stated in the literature review.

**Table 5:** The proposed models’ performance in detecting anemia using palpable palm

S/N	Algorithm	Palm (%)				
		**Accuracy**	**F1-Score**	**AUC**	**Precision**	**Recall**
1	**CNN**	**99.92**	99.89	99.95	99.79	99.98
2	**Naïve Bayes**	**99.96**	99.97	99.98	99.97	99.93
3	**Decision Tree**	**99.29**	98.97	99.38	98.77	99.18
4	**k-NN**	**99.92**	99.89	99.98	99.79	99.92
5	**SVM**	**96.34**	94.59	98.97	95.99	93.23

**Table 6:** Comparison of results with earlier studies

Reference	Algorithm Used	Results Obtained
Peksi et al*.* [13]	Naïve Bayes	90%
Tamir et al*.* [6]	SVM	78.90%
Irum et al*.* [16]	SVM	78.90%
Delgado-Rivera et al*.* [17]	CNN	77.58%
Magdalena et al*.* [19]	CNN	94%
Jain et al*.* [28]	ANN	97%

### Analysis of the machine learning algorithms performances comparatively

To evaluate the performance of the machine learning algorithms, F1-score, AUC, precision, and recall were considered approaches for evaluating the models. To avoid the occurrence of overfitting of the model's effectiveness in detecting anemia, we employed tenfold cross-validation to validate the datasets before testing the models with the datasets. The results obtained by the models for the detection of anemia are remarked. The Naïve Bayes attained 99.96% accuracy which is the highest among all the models, while the k-NN and the CNN both had an accuracy of 99.92% each. The Decision Tree also attained 99.29% accuracy. The SVM had the least accuracy of 96.34% among all the models, however, its performance is encouraging as it achieved higher accuracy than the previous studies' results.

The results obtained by the models performed significantly higher due to the augmentation of the images. In the studies by [19, 28] the authors justified that the results of their studies performed better when the images (datasets) were augmented to increase the dataset since a small data size might cause overfitting [28]. Moreover, when the results of this study were compared to the results obtained by [6, 16, 17] which were not higher due to the reason that small size of data can cause overfitting, and for that reason, the models were not trained with more datasets. Also, data augmentation was not utilized to increase the data size.

## Conclusion and future works

We compared the performance of CNN, k-NN, Decision Tree, Nave Bayes, and SVM in detecting anemia using the palm in this study. We used a primary dataset of 527 and augmented the size of the datasets to 2635 using the image augmentation technique to avoid overfitting, which is commonly caused by using small datasets. All of the proposed models used in the study produced or achieved significant results, with the Naïve Bayes attaining 99.96% accuracy which is the highest among all the models, as the k-NN and the CNN both had an accuracy of 99.92% each, with the Decision Tree it attained 97.32% accuracy, and although the SVM had the least accuracy of 94.94% among all the models its performance is encouraging.

The models were evaluated using recall, precision, F1-score, and AUC, and they were validated using tenfold cross-validation of 10% of the total datasets, while 70% and 20% of the total datasets were used for training and testing the models respectively. Based on the outcome of this study it is evident the pallor palm is a significant spot to detect anemia as also indicated by [32] in their study, especially with the use of a non-invasive method. Chand et al*.* (2017) indicated in their study that the Palm had an accuracy higher than the conjunctiva and the fingernails when the palpable palm, colour of the fingernails and the conjunctiva of the eyes were used to detect anemia in children from two months to five years. This is evidence that the palpable palm is one of the integral spots for anemia detection.

The greatest novelty of this work is the use of the palpable palm in the detection of iron deficiency anemia with its significant results, that is, the higher performance of the models since most of the previous studies used the conjunctiva of the eyes. Also, the palm is easy to be assessed as compared to the conjunctiva of the eyes, which is difficult to get access to the region of interest, especially for children below six years whose eyes sometimes may be exposed to falling objects. Also, minors’ eyes would be opened to take a picture or physically examine the conjunctiva of the eyes. There is a risk of someone’s finger entering the eye.

This could be a possible source of infection. Moreover, this study has proven that the palpable palm gives higher accuracy than the conjunctiva of the eyes when anemia is detected. In addition, a novel conceptual framework of the methodology has been created which can aid in future studies to detect anemia. The authors of this study have also created a primary dataset to detect anemia using the palpable palm images and the datasets have been published in the Medley dataset repository. Finally, this study compared the performance of five [5] machine learning algorithms and determined the algorithm with the best performance as compared to previous studies whereby only one algorithm was used.

In the future, we would retain the same machine learning algorithms or models used in this study by combining the palpable palm, conjunctiva of the eyes and fingernail images as a means of detecting anemia. This would assist us to compare and know which of the models performs better when all three features, that, palm conjunctiva of the eyes and the fingernails are combined to detect anemia.

## Supplementary Information

**Additional file 1.** Supplemental Document for Formulas.
