# Supplementary material for: Detection of iron deficiency anemia by medical images: a comparative study of machine learning algorithms
Source: BioData Min. 2023 Jan 24;16:2. doi: 10.1186/s13040-023-00319-z (PMC9875467; doi:10.1186/s13040-023-00319-z)
Supplement: Supplementary file 1 — Additional file 1. Supplemental Document for Formulas. [file 13040_2023_319_MOESM1_ESM.docx]

A mathematical model for the CNN is represented as:

$$z=w^{T}\cdot x\cdot b\ldots\ldots\ldots\ldots\ldots\ldots\ldots\ldots eqn. (1)$$

As x is the input, w is the weight, and b is the bias. The proportion chosen at random to begin the matrix = w. The back-propagation rule, on the other hand, has the mathematical model denoted as;

$$f^{'}\left( x \right)= \left( 1+e-x \right)-1\left[ 1-\left( 1+e-x \right)-1 \right]\ldots\ldots\ldots\ldots\ldots\ldots\ldots. eqn. (2)$$

$$f^{'}\left( x \right)= sigmoid\left( x \right)\left[ 1-sigmoid\left( x \right) \right]\ldots\ldots\ldots\ldots.. eqn. \left( 3 \right)$$

$$\frac{\partial O}{\partial Z2}= \left( O \right)\left( 1-O \right)\ldots\ldots.. eqn. \left( 4 \right)$$

In mathematics, Naïve Bayes is seen as;

$$P\left( c/x \right)=\frac{P\left( x/c \right)P\left( c \right)}{P\left( x \right)}\ldots\ldots\ldots\ldots\ldots\ldots\ldots\ldots eqn. (5)$$

as; $P\left( c \right)$ represents the Prior Probability of the class, $P\left( x \right)$ represent the Prior Probability of the Predictor, $x\left( c \right)$ represent the likelihood and $P$ represent the Posterior probability.

The Decision Tree is mathematically expressed as;

$$H\left( s \right)=[-P \log2 \left( P+ \right)-[-P \log2 (P-)]\ldots\ldots\ldots\ldots\ldots\ldots..eqn. (6)$$

As; $(P+)$ represents the percentage of the positive class, $\left( P- \right)$ represent the percentage of the negative class and $P$ represent the probability. The Information Gain formula also as;

$$Gain\left( S,A \right)=H\left( s \right)\sum_{/s/}^{/Sv/} H\left( Sv \right)\ldots\ldots\ldots\ldots\ldots\ldots\ldots. eqn. (7)$$

The mathematical notation for the k-NN algorithm is as follows:

$ⅆ\left( x,x^{'} \right)=$ $\sqrt{\left( x,-x_{1} \right)^{2}+div +\left( x_{n}-x_{n}^{'} \right)^{2}}\ldots\ldots\ldots\ldots\ldots\ldots\ldots.. eqn. (8)$

as the input $x$ is assigned to the maximum possible value of the class

$P\left( y=\left. j \right|x=x \right)=$ $\frac{1}{k}\sum_{i\in A} I\left( y^{\left( i \right)}=j \right)\ldots\ldots\ldots\ldots\ldots eqn. (9)$

A hyperplane is represented by a vector weight (w) and a bias (b) that are parameterized as shown by the equation;

$$w\cdot x+b=0\ldots\ldots\ldots\ldots\ldots\ldots\ldots.. eqn. \left( 9 \right)$$

$$w\cdot x+b=-1\ldots\ldots\ldots\ldots\ldots\ldots\ldots.. eqn. \left( 10 \right)$$

$$w\cdot x+b=0\ldots\ldots\ldots\ldots\ldots\ldots\ldots.. eqn. \left( 11 \right)$$

The hyper-plane function is emitted for the datasets used for training and testing the model to be categorized as;

$$f\left( x \right)=sign \left( w\cdot x+b \right)\ldots\ldots\ldots\ldots\ldots\ldots\ldots. eqn. \left( 12 \right)$$

If the kernel function is used, the above-mentioned function could be stated as;

$f\left( x \right)=sign$ $\left( \sum_{i=1}^{N} a_{i}y_{i}k\left( x_{i},x \right)+b \right)\ldots\ldots\ldots\ldots\ldots\ldots eqn. (13)$

The Equations beneath are the mathematical models for the CIE L*a*b* colour space demonstration.

$$L^{*}=116f\left( \frac{Y}{Yn} \right)-16, \ldots\ldots\ldots\ldots\ldots\ldots\ldots\ldots\ldots\ldots. eqn. (14)$$

$a^{*}=500\left\{ f\left( \frac{X}{Xn} \right)-f\left( \frac{Y}{Yn} \right) \right\}, \ldots\ldots\ldots..\ldots\ldots eqn. ($15)

$$b^{*}=200\left\{ f\left( \frac{X}{Xn} \right)-f\left( \frac{Z}{Zn} \right) \right\}, \ldots\ldots\ldots\ldots..\ldots\ldots eqn. (16)$$

Where $f\left( s \right)=s^{\frac{1}{3}}$, for $s>0.008856$

And $f\left( s \right)=7.787s+\frac{16}{116},$ for $s\leq0.008856$

The colour difference $\Delta E$ between two colours in the CIE L*a*b* (CIELAB) colour space is;

$$\Delta E=\sqrt{\left( L_{2}^{*}-L_{1}^{*} \right)^{2}+\left( a_{2}^{*}-a_{1}^{*} \right)^{2}+\left( b_{2}^{*}-b_{1}^{*} \right)^{2}}\ldots\ldots\ldots\ldots\ldots\ldots\ldots\ldots\ldots\ldots\ldots. eqn. \left( 17 \right)$$

Mathematical Models for the Image Augmentations;

The mathematical model for rotation image augmentation is stated in the equations below;

The rotation angle 𝜃 for the equations for the new coordinates of a pixel are defined as:

𝑥′=𝑥cos𝜃−𝑦sin𝜃 ………………………… eqn. (18)

𝑦′=𝑥sin𝜃+𝑦cos𝜃 …………………………. eqn. (19)

in an anti-clockwise rotation. In an analogous formulation, the position of every pixel (𝑥′, 𝑦′) in the new image can be written as a vector and the rotation matrix:

$\begin{matrix} x' \\ y' \end{matrix}$ $\left[ \begin{aligned} x^{'} \\ y^{'} \end{aligned} \right]=\left[ \begin{matrix} \cos\theta& -\sin\theta\\ \sin\theta& \cos\theta\end{matrix} \right]\left[ \begin{aligned} x \\ y \end{aligned} \right]$ …………………………….. eqn. (20)

The rotation $\left( x_{0},y_{0} \right)$ is the coordinates $(0, 0)$ point of derivation which rotates at the mid-point of the image or around a certain point. For an image or a pixel to rotate around a point $\left( x_{0},y_{0} \right)$subjectively the equation is expressed as;

$$x^{'}=x_{0}+\left( x-x_{0} \right)\cos\theta+\left( y-y_{0} \right)\sin\theta\ldots\ldots\ldots\ldots\ldots\ldots.. eqn. (21)$$

$y^{'}=y_{0}+\left( x-x_{0} \right)Sin \theta+\left( y-y_{0} \right)\cos\theta\ldots\ldots\ldots\ldots\ldots\ldots.. eqn. ($22)

The pixel at the position $\left( x_{0},y_{0} \right)$ is the only pixel that does not move from its position. If an additional image is intended from the original image one, $x and y$ then describe the equation which needs to be solved.
